# On-Campus Nursing Training During COVID-19 in Japan: A Systematic Literature Review

**DOI:** 10.7759/cureus.49479

**Published:** 2023-11-27

**Authors:** Naomi Akiyama, Shihoko Kajiwara, Atsushi Matsunaga, Kenshi Hayashida, Tomoya Akiyama

**Affiliations:** 1 Graduate School of Nursing, Nagoya City University, Nagoya, JPN; 2 School of Nursing, Gifu University of Health Science, Gifu, JPN; 3 Department of Public Health Nursing, Tohoku University Graduate School of Medicine, Tohoku University, Sendai, JPN; 4 Department of Medical Informatics and Management, University Hospital, University of Occupational and Environmental Health, Kitakyushu, JPN; 5 Center for Postgraduate Clinical Training and Career Development, Nagoya University Hospital, Nagoya, JPN

**Keywords:** japan, clinical practice, communication, education, nursing student

## Abstract

During the coronavirus disease 2019 (COVID-19) pandemic, nursing education in Japan recommended the implementation of on-campus practical training as a substitute for hospital-based clinical training. This study explores nursing students’ experiences with on-campus nursing training as an alternative to clinical practice by clarifying its advantages and disadvantages. This review followed the Preferred Reporting Items for Systematic Reviews and Meta-Analysis and we included 26 articles in this study. Our findings reveal both the advantages of acquiring nursing knowledge, basic skills, and membership among students. Contrastingly, many students failed to acquire skills related to patient communication and flexible nursing care. Thus, despite some strengths, on-campus training is not a complete replacement for clinical training. Although this method has benefits in emergency situations, it is desirable to consider other educational methods in preparation for future pandemics.

## Introduction and background

In December 2019, China reported a cluster of cases of pneumonia. Subsequently, based on the alarming levels of infection cases, in March 2020 the World Health Organization declared coronavirus disease 2019 (COVID-19) as a pandemic [1].

Globally, human lives have drastically changed since the outbreak of COVID-19, and those of nursing students are no exception. In general, students have been impacted by different sociodemographic factors, such as financial problems and low grades; rising concerns about issues related to their future professional careers and studies; as well as experiences of boredom, anxiety, and frustration caused by the lack of social interactions during the pandemic [2].

With the emergence of COVID-19, online education has gained prominence as a means for students to obtain learning and teaching. Online teaching has led to a shift in teaching theory from face-to-face interactions to those involving online/remote teaching and learning [3]. Nursing schools are being encouraged to develop contingency plans, including expanded use of simulation, telehealth, and virtual reality, lest there occur any future restrictions on clinical placements; these plans are to be implemented in keeping with the best practices and guidelines from state boards of nursing and other regulatory bodies [4].

Nursing schools have been struggling to maintain the quality of education during the pandemic due to the threat of an infectious virus spreading across global society. The pandemic has caused a hindrance to clinical practice experience, leading to nursing students’ inability to obtain the required experience in a clinical setting. For example, the United Kingdom reported that clinical placements of most junior nursing students were postponed due to an imminent shortage of supervisory staff and rapid changes in the clinical environment [5]. In the United States, all health professional institutes, including nursing schools, were asked early on to lend support to the robust public health response to COVID-19 and nursing schools halted their on-site course delivery and redesigned programs to attenuate infection risks to students and faculty [6]. To address the issue of clinical care institutions’ nonacceptance of nursing students, the Ministry of Education, Culture, Sports, Science, and Technology and the Ministry of Health, Labor, and Welfare, which control nursing training institutions in Japan, recommended the implementation of on-campus practical training as a substitute for clinical training in a hospital [7]. This study aimed to examine nursing students’ experiences of on-campus training as an alternative to clinical practice in Japan and identify the advantages and disadvantages of this substitute training approach.

## Review

Material and methods

Design

This review followed the Preferred Reporting Items for Systematic Reviews and Meta-Analyses (PRISMA) guidelines, which consist of a 27-item checklist designed to enhance the transparency of research reporting [8]. The PRISMA statement is intended to ensure comprehensive and clear documentation of research findings [8]. The accompanying flow diagram visually represents the progression of information through the various phases of a systematic review. Although PRISMA is primarily tailored for reporting reviews that assess the effectiveness of interventions, it can also serve as a valuable framework for reporting systematic reviews with objectives beyond the evaluation of intervention.

Search Method

In this study, a systematic literature review was conducted. For the systematic literature review, Ichushi-Web, a common journal search database in Japan, was searched. This database contains 400,000 articles from 4,000 journals in medical, dental, pharmacological, and nursing fields. Moreover, an additional search was conducted using Google Scholar (in Japanese) to supplement the articles from university journals. Articles published in 2020 and 2021 whose full text could be obtained from the databases were included for the review. The following search terms were used: “COVID-19,” “coronavirus,” “on-campus training,” “substituting practice,” “nursing student,” and “nursing education.”

Inclusion and Exclusion Criteria

We searched primary research articles published in Japanese between 2020 and 2021 (during the COVID-19 pandemic) on September 24, 2022. A total of 175 manuscripts were identified from the Ichushi-Web database. Manuscripts published on or before 2019 that were not classified as nursing original papers were excluded. This study aimed to investigate the experiences of nursing students with on-campus nursing training as an alternative to clinical practice in Japan during the COVID-19 pandemic. When reading abstracts of manuscripts, we excluded those that did not focus on on-campus nursing training as an alternative to clinical practice.

In addition, we excluded manuscripts that did not address accruing skills and knowledge. References for the acquisition of skills and knowledge were drawn from two key sources as follows: the “achievement goals for graduation in bachelor’s degree nursing education” [9] and the “model core curriculum for nursing education in Japan” [10]. The “achievement goals for graduation in bachelor’s degree nursing education” serve as a framework to ensure the quality of baccalaureate nursing education. This guideline outlines essential nursing practical abilities, graduation goals, educational content, and desired learning outcomes for students in the baccalaureate program.

Additionally, the “model core curriculum for nursing education in Japan” was consulted as a reference. This model core curriculum provides a comprehensive framework for nursing education in Japan, extracting core content that all nursing universities must address in their bachelor’s degree programs. It also enumerates learning objectives that serve as a reference for constructing curricula in each university. In total, this review included 15 manuscripts sourced from the Ichushi-Web database.

Japanese nursing manuscripts are often featured in bulletins published by distinct faculties. Each faculty bulletin is available not only on article search systems such as Ichushi-Web but also on Google Scholar. Thus, we also searched Google Scholar to read the abstracts of these manuscripts and added them if they were relevant to our aims. Ultimately, 26 articles were incorporated from Google Scholar.

Search Outcomes

**Figure 1:**
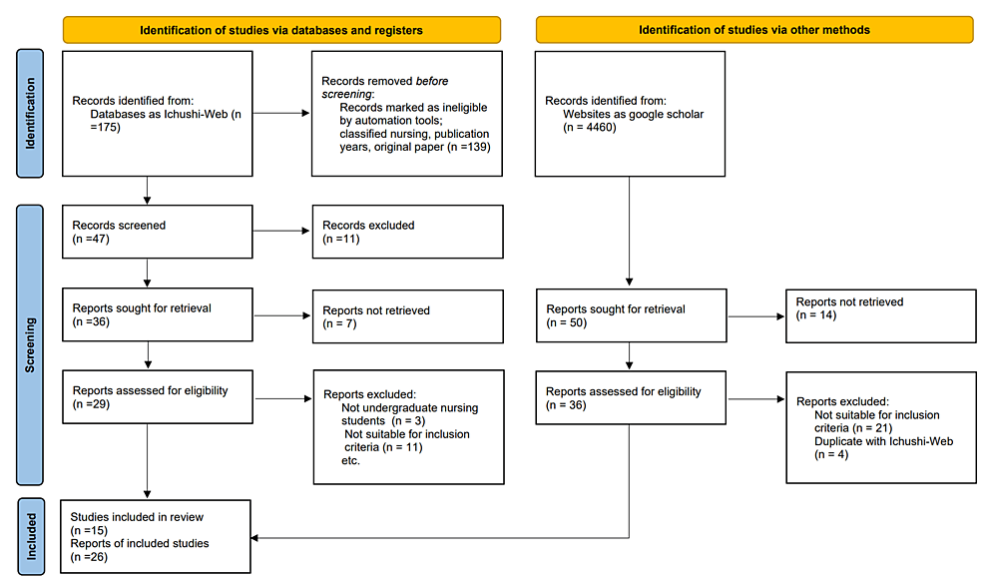
Flowchart of the selection of included studies.

Figure 1 shows a flowchart of the selection of included studies. Twenty-six articles were included for review [8].

Analysis

We employed thematic analysis, as described by Braun and Clarke [11] to identify recurring themes and reporting patterns within the manuscripts [12,13]. Thematic analysis is a valuable method for examining previous research studies. The specific reporting patterns we focused on pertained to the educational content, teaching tools, and the acquisition or lack thereof of skills during on-campus training as an alternative to clinical practice amidst the COVID-19 pandemic.

Regarding teaching activities, we systematically sought and coded descriptions detailing the tools and materials used for instructing nursing students. The codes were interpreted with guidance from Nilson [14]. Skills, both those acquired and those not acquired, were coded with reference to the “achievement goals for graduation in bachelor’s degree nursing education” [9] and the “model core curriculum for nursing education in Japan” [10].

Quality Appraisal

The selected articles were independently evaluated by two reviewers (X1 and X2) before being included in this review to identify the advantages and disadvantages of on-campus training as a substitute for clinical practice. Discrepancies in review results were discussed by the reviewers to reach a consensus.

Results

Characteristics of the Selected Articles

Table 1 presents the 26 articles that were included in this review [15-40]. These articles were authored by Japanese researchers and published in university bulletins and not academic journals, except for one, which was published in the Council Journal [38].

**Table 1:** Main characteristics of the selected studies.

No.	Authors	Year	Study design	Participants	Practice	Practice type
1	Noami et al. [15]	2022	Questionnaire survey	32 students	Fundamentals of nursing practice 1	On-campus
2	Shinohara et al. [16]	2020	Questionnaire survey	20 students	Fundamentals of nursing practice 1	On-campus
3	Uno et al. [17]	2021	Describing	81 students	Fundamentals of nursing practice 2	On-campus
4	Suzuki et al. [18]	2021	Questionnaire survey	100 students	Fundamentals of nursing practice 2	On-campus
5	Ito et al. [19]	2021	Student report	100 students	Fundamentals of nursing practice 2	On-campus practice and limited clinical practice
6	Matsumoto et al. [20]	2020	Questionnaire survey	102 students	Fundamentals of nursing practice 2	On-campus
7	Kagawa et al. [21]	2021	Student report	51 students	Acute nursing practice	On-campus
8	Ishizuka et al. [22]	2022	Questionnaire survey	37 students	Acute nursing practice	On-campus
9	Ohtori et al. [23]	2020	Describing	91 students	Chronic nursing practice	On-campus practice and limited clinical practice
10	Nomura et al. [24]	2021	Student report	6 students	Chronic nursing practice	On-campus practice and limited clinical practice
11	Ohtori and Saito [25]	2020	Questionnaire survey, interview survey	85 students and 5 clinical indicators	Chronic nursing practice	On-campus practice and limited clinical practice
12	Shimazu et al. [26]	2021	Student report	-	Chronic nursing practice	On-campus
13	Tabata et al. [27]	2020	Questionnaire survey	82 students	Gerontological nursing practice	On-campus
14	Kinoshita et al. [28]	2022	Student report	58 students	Gerontological nursing practice	On-campus
15	Kimura et al. [29]	2020	Describing	80 students	Gerontological nursing practice	On-campus
16	Uchino and Horiuchi [30]	2021	Describing	81 students	Gerontological nursing practice	On-campus
17	Okada et al. [31]	2021	Questionnaire survey	71 students	Home nursing practice	On-campus
18	Imai et al. [32]	2022	Describing	38 students	Home nursing practice	On-campus
19	Wada et al. [33]	2021	Questionnaire survey	42 students	Home nursing practice	On-campus
20	Aoi and Bekku [34]	2020	Student report	50 students	Psychiatric nursing practice	On-campus
21	Tanaka et al. [35]	2020	Questionnaire, student report	39 students	Child nursing practice	On-campus
22	Oota et al. [36]	2021	Questionnaire, student report	80 students	Integrative nursing practice	On-campus
23	Kitamura et al. [37]	2021	Describing	18 students	Integrative nursing practice	On-campus practice and limited clinical practice
24	Honda et al. [38]	2021	Questionnaire survey	29 students	Public health nursing practice	On-campus practice and limited clinical practice
25	Watanabe et al. [39]	2020	Describing	29 students	Public health nursing practice	On-campus practice and limited clinical practice
26	Sawada and Takanami [40]	2021	Questionnaire survey	34 students and 16 teachers	Specialized nursing practice, but not identified the type of nursing practice	On-campus

Out of the 26 papers, four (15.4%) focused on fundamental nursing practice for second-grade nursing students, chronic nursing practice, and gerontological nursing practice; three focused on home nursing practice (11.5%); two focused on acute, fundamental nursing practice for first-grade nursing students, integrative nursing practice, and public health nursing practice; and one focused on psychiatric nursing practice and child nursing practice. Furthermore, 19 articles (73.1%) focused on on-campus training, whereas seven (26.9%) were about on-campus training and limited clinical practice.

Practice Content Represented As Teaching Activities

Table 2 shows the frequency of practice content in the form of teaching activities. The top 10 most frequent items were “conducting a conference, discussion, or presentation” (n = 25, 96.2%), “using hypothetical patient documented in case-based learning” (n = 20, 76.9%), “roleplaying by nursing students as patients” (n = 18, 69.2%), “using video materials to teach nursing” (n = 16, 61.5%), “roleplaying by nursing teachers as patients” (n = 11, 42.3%), “collaborating with clinical instructor” (n = 11, 42.3%), and “using patient simulators or simulated patient experience equipment” (n = 10, 38.5%), “mimicking a clinical setting” (n = 8, 30.8%), “providing individualized instructions” (n = 8, 30.8%), “recording and debriefing” (n = 6, 23.1%).

**Table 2:** Practice content as teaching methods of alternative clinical practice during COVID-19. COVID-19: coronavirus disease 2019

Practice content	Number of articles (percentage)	References
Conducting a conference, discussion, or presentation	25 (96.2%)	[15-35,37-40]
Using hypothetical patients documented in case-based learning	20 (76.9%)	[15-18,20-22,26-35,37,38,40]
Roleplaying by nursing students as patients	18 (69.2%)	[15,17-19,21-29,32,37-40]
Using video materials to teach nursing	16 (61.5%)	[15,17,18,20-22,24,28-33,36,38,40]
Roleplaying by nursing teachers as patients	11 (42.3%)	[15-18,21,22,26,27,37,39,40]
Collaborating with a clinical instructor	11 (42.3%)	[19,21,23,25,26,29-33,38]
Using patient simulators or simulated patient experience equipment	10 (38.5%)	[17,19-21,26,27,29,30,34,40]
Mimicking a clinical setting	8 (30.8%)	[15,16,18,20,22,30,32,40]
Providing individualized instructions	8 (30.8%)	[20,21,24,30,33,35,36,39]
Recording and debriefing	6 (23.1%)	[21,25,26,37,39,40]
Using a real record of clinical cases	5 (19.2%)	[19,23,25,39,40]
Walking around an area as fieldwork	3 (11.5%)	[32,38,39]
Roleplaying by nursing teachers as clinical nurses	3 (11.5%)	[21,26,39]
Using real hospital documents	3 (11.5%)	[26,32,34]
Using remote equipment to communicate with real patients	2 (7.7%)	[19,30]
Hiring a patient model	1 (3.8%)	[40]
Others	4 (15.4%)	[21,26,27,34]

Skills Acquired

Table 3 shows the frequency items of skills acquired. The number of reports describing “understanding the nursing process to plan evidenced-based nursing care” and “collaboration among student group members” was 15 (57.7%) for each and that of those on “understanding nursing and the role of its activities among various settings and team structures” was 11 (42.3%). The number of reports on the “implementation of the safety/comfort/independence of nursing care skills” was 10 (38.5%), and the number of reports on “understanding nursing practice adapted to diseases and recovery process” and “understanding patients such as physical/mental reactions associated with health problems and treatment” was eight (38.5%) for each.

**Table 3:** Skills acquired through on-campus training as an alternative to clinical training during COVID-19. COVID-19: coronavirus disease 2019

Skills acquired	Number of articles (percentage)	References
Understanding the nursing process to plan evidence-based nursing care	15 (57.7)	[15,17-22,24,25,30,33,34,37,39,40]
Collaboration among student group members	15 (57.7)	[18,22,24,25,27,30,31,33,34,36,37,39]
Understanding nursing and the role of its activities among various settings and team structures	11 (42.3)	[16,22,27,28,30-32,34,36,38,39]
Implementing the safety/comfort/independence of nursing care skills	10 (38.5)	[15,19-22,26,27,35,37,40]
Understanding nursing practice adapted to diseases and recovery process	8 (30.7)	[17,21,28,30,32,34,35,37]
Understanding patients such as physical/mental reactions associated with health problems and treatment	8 (38.5)	[15,24,28,30,32,34,35,39]
Learning student’s own characteristics related to basic attitude, how to form a team, and nursing practice	5 (19.2)	[24,26,29,34,36]
Understanding nursing practice in various settings such as hospitals or facilities	3 (11.5)	[16,22,30]
Understanding discharge planning and continued nursing among various settings and team structures	3 (11.5)	[22,29,37]
Advocation for dignity and human rights such as considering clinical ethics issues	3 (11.5)	[15,30,34]
Knowledge of multi-professional collaboration among various settings and team structures	2 (7.7)	[31,33]
Correctly addressing specific health problems and imagining meeting patients’ wishes	2 (7.7)	[26,35]

Skills Not Acquired

Table 4 shows the frequency items of skills not acquired. The number of reports on “communicating patient responses” was 11 (42.3%) and that of those on “implementing safety/comfort/independence in nursing cares” was 10 (38.5%). Further, there were seven reports (26.9%) each on “collecting patient information regarding nursing practice adapted to the disease and recovery process and planning evidence-based nursing care” and “planning nursing practice in various settings such as hospitals or facilities.” Additionally, “understanding patients’ physical/mental reactions associated with health problems and treatment” was reported in six articles (23.1%).

**Table 4:** Skills not acquired through on-campus training as an alternative to clinical practice during COVID-19. COVID-19: coronavirus disease 2019

Skills not acquired	Number of articles (percentage)	References
Communicating patient responses	11 (42.3)	[17-22,25,27,35,36,39]
Implementing safety/comfort/independence in nursing cares	10 (38.5)	[17,18,20,26,32-36,38]
Collecting patient information regarding nursing practice adapted to the disease and recovery process and planning evidence-based nursing care	7 (26.9)	[17,18,20,21,25,26,33]
Planning nursing practice in various settings such as hospitals or facilities	7 (26.9)	[16,20,30-33,35]
Understanding patients’ physical/mental reactions associated with health problems and treatment	6 (23.1)	[17,21,22,31,37,40]
Building supportive human relationships	5 (19.2)	[18,20,25,30,34]
Multi-professional collaborations in multiple settings and team structures	2 (7.7)	[20,24]
Understanding the role of nursing and its activities in multiple settings and team structures	1 (3.8)	[16]
Advocacy of dignity and human rights	1 (3.8)	[24]
Taking care of multiple patients	1 (3.8)	[36]
Flexibility in taking care of patient's condition	1 (3.8)	[20]
Quick assessment	1 (3.8)	[20]
Providing evidence-based nursing care according to the needs of individual patients	1 (3.8)	[35]

Discussion

Clinical practice involves a lot of learning, but it is necessary to consider the burden on both students and clinical instructors. Nursing students who received clinical practice training in actual clinical settings during the COVID-19 pandemic have some practical concerns, including the provision of fewer learning opportunities and a lack of complete understanding of why they chose to become a nurse [41]. In October 2021, the Ministry of Education, Culture, Sports, Science, and Technology of Japan surveyed schools for training public health nurses, midwives, and other such practitioners and reported that 97.2% of the colleges provided some kind of practical training as an alternative to clinical training [42]. Practical training as an alternative to clinical training can be conducted through on-campus, online (interactive communication is possible), theoretical, report, and on-demand (one-way communication) training.

Our findings suggested similar trends to those found in ministry reports, such as using documented patient records and roleplay by nursing students or teachers. Owing to limited time and manpower due in Japan to COVID-19, training schools provided only on-campus training for nursing students. This study identified the advantages and disadvantages of on-campus training as a substitute for clinical practice by conducting a systematic literature review. The study results indicate that nursing educators implemented various on-campus training programs, which partly substituted clinical practice but led to challenges as well. On the one hand, the advantages of on-campus training included students’ ability to acquire knowledge and basic skills and student group memberships; on the other hand, students could only partly acquire problem-solving skills and faced difficulties in patient-response-based communication.

Acquiring Nursing Knowledge, Basic Skills, and Student Group Memberships

The study revealed that students could acquire knowledge and basic nursing skills in terms of knowledge of the processes and diseases through on-campus training. However, regardless of the type of practice content, such nursing skills could only be partly acquired. In on-campus training, students learned about patient-related diseases and acquired knowledge of nursing patients at their own pace [17,21,28,30,32,34,35,37,40]. Through a comparison of students’ understanding of the nursing process before and after on-campus training, it was revealed that a higher rate of students understood these aspects after practical clinical practice as compared with on-campus training [15,17-22,24,25,30,33,34,37,39,40]. In terms of practice content, via the use of a simulator, nursing students could understand the patient’s disease and obtain knowledge about the problem-solving process [30,34]. However, these skills can be acquired not only through the use of a simulator but also through role-playing exercises where students or teachers act as patients [17,21]. Simulation is an activity or technique to produce an experience without going through a real event. Simulation activities are typically followed by a debriefing to facilitate reflection, learning, abstraction, conceptualization, and connections to real events [43]. Simulation featuring live models provides support for the acquisition of complex skills. However, to use simulators for training, sufficient and skilled instructors are necessary, as well as several simulators. In Japan, a simulator, which means a simulated human model, is extremely expensive, and therefore, numerous models cannot be purchased. The use of virtual patients, which are interactive digital simulations of clinical scenarios, is a more effective way to acquire knowledge and improve skills [44]. However, the effective utilization of virtual patients might pose challenges in on-campus training, including issues pertaining to staffing and teaching techniques.

Clinical training in hospitals makes nursing students feel anxious and can burden them. This is because it is an unfamiliar environment where they have the responsibility of providing care to real patients. In contrast, as on-campus training is held on the campus of nursing training institutions, the students felt relaxed, had enough time, and received training for shorter durations compared with clinical practice training at hospitals [21-27]. In the on-campus training environment and surrounded by peers, they were able to feel some sort of kinship among student groups [18-22,24,27,30,31,33,34,36,37,39]. However, training in this familiar environment caused them to lose their sense of tension [15]. Unfortunately, in clinical settings, there is a fear of student-to-patient transmission during the pandemic, which has made it difficult for the introduction of multiple students into patient care. As a result, students lose the opportunity to observe patient care provided by nurses/other students in an actual clinical setting. However, on-campus training provides an opportunity to enhance collaboration among students.

Problems Associated With On-Campus Training: Patient-Response-Based Communication and Flexibility of Nursing Care

Our study findings revealed that patient response-based communication is difficult to simulate in on-campus training [17-22,25,27,35,36,39]. However, through this training, nursing students can partially master the skills of nursing and patient communication. Moreover, there are some effective approaches for learning, such as using documented patient records and/or simulator scenarios [15-35,37,38,40]. Gutiérrez-Puertas et al. conducted a systematic review that showed that communication skills can be improved through interventions such as simulation, which includes roleplaying [45]. However, when nursing students roleplay as patients, their behaviors are docile, and they exhibit predictable patient movements. No colorful ad-libs were used, especially since students who performed the role of patients have little experience in an actual clinical setting [25]. In an actual setting, the patient listens carefully to what the student says and may not provide the student with an ideal response. Thus, students find patient-response-based communication difficult without clinical practice.

For the collection of patient-related information, documented patient records are useful to nursing students for learning and understanding nursing processes. Therefore, by relying on these documents, students would not need to invest too much effort in searching and gaining information [20]. However, it is impossible to acquire the ability to select only the necessary information from huge amounts of data, such as medical charts, and examine it within the limited time allotted for clinical practice through on-campus training and not clinical training [17,18,20,21,25,26,33].

Developing an Effective On-Campus Training

Most of the studies reviewed have been performed on-campus, with no experience in actual clinical practice and only online learning. Due to the COVID-19 pandemic, nursing students were faced with the unprecedented challenges of having to abruptly shift to online learning, which, although useful, caused a lot of stress [46]. Online learning has many advantages, including learning in a flexible learning environment, academic achievements, and student-centered learning; however, some disadvantages also exist, such as low academic integrity, a rigid learning environment, and high family burden [47]. To be trained on-campus or online, an appropriate internet environment is required, along with robust online troubleshooting by nursing teachers. Although nursing teachers made various efforts to implement alternative training for clinical placement, many students were anxious about not having the opportunity to obtain experience in an actual clinical setting [16,27]. Nursing students’ unhappiness due to the limited clinical educational experiences was brought about by COVID-19 [48].

In total, 11 studies reported on “collaborating with clinical settings” [19,21,23,25,26,29-33,38]. Ohtori et al. examined the collaboration between clinical instructors such as hospital nurses and teachers, and they reported that clinical instructors cared for real patients, used nursing students’ nursing plans, and could offer insights to students [23]. Honda et al. also studied the collaboration between nursing students and clinical instructors such as public health nurses and teachers, they reported that nursing students first analyzed clinical cases and then telephoned clinical instructors to directly gain new information and insights from them [38]. Due to collaboration with instructors in clinical settings, nursing students were able to obtain an impression of their future role as nurses and nursing activities.

Study Limitations

This study clarified nursing students’ experiences with on-campus training as an alternative to clinical placement for Japanese nursing students during the COVID-19 pandemic, showing which skills could and could not be acquired during the process. However, there were a few limitations. First, 25 out of the 26 articles included for the review in this study were published in university bulletins and not in academic journals; thus, not enough material was available for investigating the advantages or disadvantages of on-campus training for nursing students. In addition, since our review included many studies that employed descriptive analysis, we were unable to treat “skills acquired” and “skills not acquired” as exclusive events; thus, the descriptions in Tables 3, 4 are similar.

However, due to the ongoing COVID-19 pandemic situation worldwide, nursing education has faced dramatic changes, and more time is needed to verify the benefits of on-campus training through interventional studies. Second, only Japanese-language studies were investigated since English-language studies regarding on-campus training for Japanese nursing students could not be found.

## Conclusions

Nursing students experienced training in school and/or home as an alternative to clinical placement during the COVID-19 pandemic. Our findings reveal both the advantages, such as acquiring nursing knowledge, basic skills, and membership among student groups, as well as disadvantages of on-campus training, such as the problem of patient-response-based communication and flexible nursing care. Although nursing educators made various efforts to implement alternative training as a substitute for clinical placement, many students were anxious about missing the experience of a clinical setting and the fact that they could not interact with patients. Although some articles reported that the collaboration between clinical preceptors and nursing schools helped students visualize clinical settings and patients, more such collaborations are needed to enhance nursing training.
